# Phosphate and Phosphate Transporters in Polycystic Kidney Disease

**DOI:** 10.34067/KID.0000001274

**Published:** 2026-07-30

**Authors:** Vicente E. Torres, Xiaofang Wang, Christian Hanna

**Affiliations:** 1 Division of Nephrology and Hypertension, Mayo Clinic, Rochester, Minnesota; 2 Robert M. and Billie Kelley Pirnie Translational PKD Center, Mayo Clinic, Rochester, Minnesota; 3 Division of Pediatric Nephrology and Hypertension, Mayo Clinic, Rochester, Minnesota

**Keywords:** ADPKD, CKD

Maintenance of inorganic phosphate (Pi) levels within narrow ranges is essential for normal cellular metabolism. Highly regulated renal excretion and less regulated intestinal absorption maintain body Pi balance. When the number of functioning nephrons is reduced in CKD, Pi retention stimulates the production of phosphaturic parathyroid hormone (PTH) and fibroblast growth factor 23 (FGF23). PTH and FGF23 inhibit Pi reabsorption in the proximal tubule and increase Pi fractional excretion and intratubular concentration in residual nephrons. When this compensatory mechanism cannot keep up with Pi intestinal absorption, Pi concentration in extracellular fluid increases. While hyperphosphatemia promotes inflammation, oxidative stress, and cardiovascular disease, high Pi concentrations in tubular fluid may be toxic and contribute to CKD progression.

Two families of sodium-dependent Pi transporters, NaPi2a, NaPi2b, and NaPi2c, coded by *SLC34A1*, *SLC34A2*, and *SLC34A3*, and PiT1 and PiT2, coded by *SLC20A* and *SLC20B*, and the xenotropic and polytropic retrovirus receptor 1 (XPR1), the only cellular Pi exporter, are critical for Pi homeostasis. NaPi2a, NaPi2c, and PiT2 (in renal proximal tubules) and NaPi2b (in small intestine and Henle's loops), under the control of PTH, FGF23, and other factors, balance intestinal absorption and renal excretion, and maintain the extracellular Pi concentration within a narrow range (0.7–1.6 mM in adults). PTH and FGF23 promote endocytosis and degradation of NaPi2a and NaPi2c; FGF23 also downregulates their transcription. In all cells, intracellular Pi concentration, approximately 5 mM, is maintained by NaPi2b and PiT1-mediated Pi entry and by XPR1 mediated export, and by rates of Pi incorporation into and release from organic compounds.

Acute Pi loading in rodents and humans induces elevations of PTH and FGF23 within minutes, massive phosphaturia, intratubular precipitation of calcium Pi, and kidney injury. Susceptibility to Pi nephrotoxicity varies among species (rats more than mice) and sexes (females more than males). Parathyroidectomy prevented renal deposition of calcium Pi in the rat. Estrogens transcriptionally downregulate renal *Slc34a1* and *Slc34a3* and upregulate intestinal *Slc34a2*, which may account for increased susceptibility of females to Pi toxicity.^1,2^

Moderate increases in dietary Pi and CKD stimulate the PTH and FGF23 secretion, suppress NaPi2a and NaPi2c mediated Pi reabsorption in proximal tubules, and increase Pi fractional excretion and intratubular concentration. Animal studies and some clinical observations support that PTH, FGF23, and phosphaturia may contribute to the progression of CKD.^3^

The elegant study by Jansson *et al.* in the current issue of *Kidney360*^4^ strongly suggests that increased phosphaturia may contribute to cyst formation and growth in early autosomal dominant polycystic kidney disease (ADPKD), in addition to CKD progression at advanced stages. This study in *Pkd1*^RC/RC^ and wild-type mice extends their previous observation that Pi restriction attenuates cystic kidney disease in *pcy*/*pcy* mice. Nonphytate 1.1% Pi diet induced marked deposition of calcium Pi crystals, mineral deposition quantified by microcomputed tomography, and aggravation of the cystic disease, with sexual dimorphism (more severe in females), in *Pkd1*^RC/RC^ mice. No calcium Pi deposition was detected in mice fed nonphytate 0.2% Pi diet. *Slc34a1*^−/−^ mice lacking proximal tubule NaPi2a were used to directly asses the role of urinary Pi, eliminating confounders such as high circulating concentrations of phosphaturic hormones, hyperphosphatemia, or Pi content of the diet. Elimination of *Slc34a1* induced dispersed calcium Pi crystals and mild tubular dilation in wild-type, and marked calcium Pi deposition and aggravation of the cystic disease in *Pkd1*^RC/RC^ mice. The effect of eliminating *Slc34a1* was not sexually dimorphic, suggesting that estrogen mediated downregulation of *Slc34a1* contributes to the sexual dimorphic effect of a high Pi diet. Unexpectedly, FGF23 levels were lower in *Pkd1*^RC/RC^ mice on a high Pi diet or lacking *Slc34a1* compared with their respective controls. The results of these studies are consistent with the aggravation of polycystic kidney disease in PCK rats on a high Pi diet^5^ (Wang *et al. J Am Soc Nephrol.*, 23-S: 948A, 2012, abstract). In the PCK rats, however, high Pi diet was associated with higher FGF23 levels, possibly because different genes are mutated in the two models. The results of Jansson *et al.* are also consistent with the development of kidney cysts in patients with hereditary hypophosphatemic rickets with hypercalciuria, a rare monogenic disorder caused by *SLC34A3* pathogenic variants.^6^

Jansson *et al.* propose that intratubular precipitation of calcium Pi crystals promotes cystogenesis and aggravates PKD, without ruling out the possibility that high concentrations of urinary Pi alone could be toxic to tubular epithelial cells. The crystalluria hypothesis is supported by a previous study suggesting that the mechanical effects of calcium oxalate crystals in male Han: Sprague-Dawley cy/+ rats treated with ethylenglycol, or of calcium Pi crystals in PCK rats fed a high Pi diet, activated mammalian target of rapamycin, caused tubular dilation, and aggravated the cystic disease.^5^ Another study has shown that tethering of calcium Pi crystals to toll-like receptor 4 on the surface of proximal tubule cells against tubular flow facilitates their endocytosis and induce disturbed endosomal trafficking and tubule cell damage.^7^ In this study, mice lacking toll-like receptor 4 were resistant to crystal-induced tubular injury.

Jansson *et al.* focused on NaPi2a because it is the most abundant renal Pi transporter and mediates ≈70% to 80% of active Pi transport in mice (NaPi2c also plays an important role in humans). Nevertheless, recent studies showed increased expression of NaPi2b in oxalate induced kidney disease where NaPi2a and NaPi2c were downregulated and in many types human CKD, not only in Henle's loops but also in collecting ducts and other tubular segments (Busch *et al. Nephrol Dial Transplant.* 38-S1, 2023 doi:10.1093/ndt/gfad063b_5592, abstract; Hu *et al. Nephrol Dial Transplant.* 38-S1, 2023; doi:10.1093/ndt/gfad063c_2685, abstract).^8^ Consistent with these observations, we found increased expression of *Slc34a2* and NaPi2b and reduced expressions of *Xpr1* and *Kidins220* (a partner protein required for proper cellular localization and activity of XPR1), in *Pkd1*^RC/RC^ mice and mice with constitutive activation of protein kinase A in the distal nephron and collecting ducts, compared with wild-type mice (unpublished data). *Slc34a2* mRNA and NaPi2b protein were also overexpressed in PCK rats fed a high compared with a normal Pi diet (unpublished data). The role of these Pi transporters has been extensively studied in cancer, but not in PKD. Upregulation of NaPi2b and downregulation of XPR1 and Kidins222 may increase intracellular Pi, thus providing Pi to proliferative cells and promoting proliferation,^9^ or result in toxic accumulation of intracellular Pi and induce apoptosis, thus providing a therapeutic opportunity for certain types of cancer.^10^

Very few studies have examined the effect of phosphaturia on disease progression in individuals with ADPKD. Jansson *et al.* in the current issue of *Kidney360* demonstrated extensive deposition of calcium Pi crystals in end-stage ADPKD compared with normal kidneys but did not include end stage kidneys of other etiologies. The Consortium for Radiologic Imaging Studies of Polycystic Kidney Disease study showed that urine Pi excretion adjusted for height and baseline ln total kidney volume (lnTKV) was positively correlated with lnTKV slope in females but not in males (Irazabal *et al. J Am Soc Nephrol.*, 23-S: 703A, 2012, abstract). The Developing Intervention Strategies to Halt Progression of ADPKD study showed that ratios of tubular maximum reabsorption rate of Pi to GFR <0.8 mmol/L (defined as kidney Pi wasting) and high 24 hours urine Pi excretions (possibly reflecting increased intestinal Pi absorption) were associated with larger height adjusted kidney volume, CKD stage, rate of eGFR decline, and risk for a composite kidney outcome (≥30% eGFR decline, kidney failure or KRT), despite lower serum Pi levels.^11^ The authors concluded that Pi wasting is prevalent with ADPKD, is associated with more rapid disease progression, may be caused by early proximal tubular dysfunction, and may contribute to disease progression. This study, however, did not include individuals with other forms of CKD.

Urinary Pi excretion is mainly determined by the Pi content of the diet. Average daily Pi intake in adults is 1368 mg, twice the recommended allowance of 700 mg. Whether moderate increases in phosphaturia accelerate ADPKD or CKD progression remains uncertain. The 2025 ADPKD and 2024 CKD Kidney Disease Improving Global Outcomes guidelines suggest using renal dietitians or accredited nutrition providers to educate affected individuals about dietary adaptations regarding sodium, phosphorus, potassium, and protein intake, tailored to individual needs, and severity of CKD and comorbid conditions. Recent strategies include limiting highly bioaccessible Pi-containing food additives, prioritizing plant (lower bioaccessibility) over animal (higher bioaccessibility) protein, and optimizing cooking techniques to reduce Pi or Pi bioaccessibility. Measurement of 24 hours urine Pi is the preferred method to approximate Pi dietary intake despite substantial day-to-day variability, and estimations of protein intake using urea excretion and the Maroni equation are rarely used in clinical practice to monitor compliance with the dietary recommendations and their safety.

In summary, the study by Jansson *et al.* strongly suggests that excessive phosphaturia aggravates PKD in an orthologous model and possibly in humans with ADPKD. More research is needed to determine the underlying mechanism, the role of other Pi transporters in addition to NaPi2a, the clinical significance of moderate degrees of hyperphosphaturia, and the value of implementing and monitoring Pi controlled diets.
